# *In vitro* and *in vivo* Expression of Interstitial Collagenase/MMP-1 by Human Mast Cells

**DOI:** 10.1155/2000/82708

**Published:** 2000

**Authors:** Nick Di Girolamo, Denis Wakefield

**Affiliations:** Inflammation Research Unit School of Pathology The University of New South Wales Sydney 2052 Australia

**Keywords:** collagenase, gelatinase, mast cells, matrix metalloproteinase, TIMP

## Abstract

Degradation of the extracellular matrix occurs under physiological and pathological conditions, thought to be principally mediated by a family of neutral proteolytic enzymes termed the matrix metalloproteinases (MMPs). The present study was initiated to determine whether mast cells have the ability to produce these proteases in diseased and normal human tissue. Immunohistochemistry and *in situ* hybridization was performed to localize interstitial collagenase protein and mRNA transcripts in diseased human tissue. The human mast cell line HMC-1 was cultured under serum free conditions, stimulated with phorbol mystrate acetate (PMA) and supernatants analyzed by Western blotting and zymography to determine the profile of secreted MMPs. The dog mast cell line BR, known to secrete gelatinolytic enzymes, was used in parallel studies. Total RNA was extracted and analyzed by RT-PCR for the expression of tissue inhibitors of MMP (TIMPs). Collagenase-1 protein and mRNA were expressed by tryptase and chymase positive human mast cells in all tissue analyzed. This proteinase wa also detected in the cytoplasm and conditioned media of HMC-1 cells. PMA induced gelatinolytic activity in both mast cell lines examined. TIMP-1 immunoreactivity was detected and TIMP-1, and-2 (but not TIMP-3) mRNA transcripts were amplified from HMC-1 cells. This is the first demonstration of the expression of collagenase-1 by human mast cells in both inflamed and normal tissues, and by a human mast cell line. MMPs secreted by these cells could contribute to the extensive matrix lysis characteristic of diseases such as rheumatoid arthritis and inflammatory ocular disorders. Alternatively collagenase-1 production by mast cells may play a critical role in cell invasion and migration into sites of inflammation.


*Developmental Immunology*, 2000, Vol. 7(2-4), pp. 131-142
Reprints available directly from the publisher
Photocopying permitted by license only

© 2000 OPA (Overseas Publishers Association)
N.V. Published by license under the
Harwood Academic Publishers imprint,
part of the Gordon and Breach Publishing Group.
Printed in Malaysia

### *In vitro* and *in vivo* Expression of Interstitial Collagenase/MMP-1 by Human Mast Cells

NICK DI GIROLAMO* and DENIS WAKEFIELD

*Inflammation Research Unit, School of Pathology, The University of New South Wales, Sydney, 2052, Australia*

Degradation of the extracellular matrix occurs under physiological and pathological conditions, thought to be principally mediated by a family of neutral proteolytic enzymes termed the matrix metalloproteinases (MMPs). The present study was initiated to determine whether mast cells have the ability to produce these proteases in diseased and normal human tissue. Immunohistochemistry and *in situ* hybridization was performed to localize interstitial collagenase protein and mRNA transcripts in diseased human tissue. The human mast cell line HMC-1 was cultured under serum free conditions, stimulated with phorbol mystrate acetate (PMA) and supernatants analyzed by Western blotting and zymography to determine the profile of secreted MMPs. The dog mast cell line BR, known to secrete gelatinolytic enzymes, was used in parallel studies. Total RNA was extracted and analyzed by RT-PCR for the expression of tissue inhibitors of MMP (TIMPs). Collagenase-1 protein and mRNA were expressed by tryptase and chymase positive human mast cells in all tissue analyzed. This proteinase was also detected in the cytoplasm and conditioned media of HMC-1 cells. PMA induced gelatinolytic activity in both mast cell lines examined. TIMP-1 immunoreactivity was detected and TIMP-1, and –2 (but not TIMP-3) mRNA transcripts were amplified from HMC-1 cells. This is the first demonstration of the expression of collagenase-1 by human mast cells in both inflamed and normal tissues, and by a human mast cell line. MMPs secreted by these cells could contribute to the extensive matrix lysis characteristic of diseases such as rheumatoid arthritis and inflammatory ocular disorders. Alternatively collagenase-1 production by mast cells may play a critical role in cell invasion and migration into sites of inflammation.

*Keywords:* collagenase, gelatinase, mast cells, matrix metalloproteinase, TIMP

### INTRODUCTION

Recent studies have focused on the potential importance of mast cells in a variety of inflammatory disorders affecting the integrity of the connective tissue matrix such as rheumatoid arthritis (RA) (Godfrey et al., 1984) and asthma (Galli, 1993). Mast cells have been shown to play a key role in initiating inflammatory responses, via their release of pro-inflammatory mediators such as cytokines (Gordon and Galli, 1990), growth factors (Powers et al., 1997), chemokines (Wang et al., 1998; Moller et al., 1993), histamine and proteases (McNeil, 1996). Proteases such as tryptase and chymase serve as specific markers for mast cells (Schwartz, 1994). Tryptase has been shown to degrade fibronectin (Lohi et al., 1992) and chy-

* Correspondence: Dr Nick Di Girolamo, Inflammation Research Unit, School of Pathology, The University of New South Wales, Sydney, 2052, Australia. Telephone: +61–2–93852533. Fax: +61–2–93851389. Email: N.Digirolamo@unsw.edu.au mase is active against basement membrane proteins such as collagen type IV and V, laminin and fibronectin (Vartio et al., 1981). Characteristically, mast cells are localized adjacent to blood vessels and amongst resident connective tissue and epithelial cells, hence their ability to promptly influence their microenvironment upon degranulation.

Matrix metalloproteinases (MMPs) are a family of neutral proteolytic enzymes active against all components of the extracellular matrix (ECM) (Stetler-Stevenson, 1996). These enzymes are divided into four subclasses depending on substrate specificity. They include the collagenases (collagenase-1, –2, –3), which are capable of cleaving collagens types I, II, and III at a single locus. The gelatinases, which consist of gelatinase A and gelatinase B, preferentially degrade denatured collagens (gelatins) and type IV collagen (the main constituent of basement membranes). The stromelysins, which comprise stromelysin-1, –2, –3 and matrilysin, have broad substrate specificity, and are capable of digesting a number of ECM proteins such as laminin, fibronectin, vitronectin, and proteoglycans. Finally, the membrane-associated MMPs (MT-MMPs) can activate other MMPs and degrade some fibrillar collagens.

Under normal physiological conditions, the process of connective tissue remodeling by MMPs occurs under stringent control. Uncontrolled remodeling generally leads to degradative pathologies, which are usually attributed to a breakdown in the MMP regulatory mechanism. Regulation of MMPs occurs at the level of gene transcription, proenzyme activation, and inhibition via the action of specific naturally occurring tissue inhibitors of MMPs (TIMPs). Most MMPs are not constitutively expressed, but can be induced by various stimuli including; phorbol ester, growth factors, cytokines (Mauviel, 1993; Ries and Petrides, 1995) oncogene products, and cell-to-cell or cell-to-matrix interactions. The activity of MMPs on ECM proteins is inhibited by the TIMPs. TIMPs bind MMPs forming a stable noncovalent and irreversible 1:1 stoichiometric complex. In addition to their roles as MMP inhibitors, TIMPs display growth factor-like activity (Stetler-Stevenson et al., 1992) and promote apoptosis (Guedez et al., 1998).

MMPs are secreted as latent pro-enzymes which require activation in the extracellular space. In addition to their activation by organomercurials, plasmin, trypsin, chymotrypsin, neutrophil elastase, and other MMPs, it has also been demonstrated that serine proteases (derived from mast cells) are capable of activating MMPs. The ability of purified skin mast cell chymase to activate human interstitial pro-collagenase has been examined (Saarinen et al., 1994) and results have demonstrated the cleavage of pro-collagenase in a time and dose dependent manner. The mast cell tryptase-dependent activation of pro-collagenase has been shown to be dependent entirely on the activation of pro-stromelysin (Gruber et al., 1989). Despite these investigation, the precise mechanism(s) of MMP activation *in vivo* remain unclear.

Observations of the association of mast cells with areas of connective tissue destruction and MMP expression have previously been reported at sites of tumor invasion (Dabbous et al., 1986), at regions of joint destruction in RA (Tetlow and Woolley, 1995; Gotis-Graham and McNeil, 1997), in areas of matrix degradation in scleritis (Di Girolamo et al., 1998a; Di Girolamo et al., 1998b), in atherosclerotic plaques (Johnson et al., 1998; Kaartinen et al., 1998) and in the human endometrium throughout the menstrual cycle (Salamonsen and Woolley, 1996). Although the role of mast cells have been extensively studied, the role of mast cell proteinases is not entirely clear. This study was initiated to explore the capacity of human mast cells to produce proteinases which specifically degrade matrix components.

### RESULTS

### Collagenase-1 Is Expressed By Mast Cells In Diseased And Normal Human Tissue

Diseased and normal human tissue was serially sectioned and analyzed immunohistochemically to determine the capacity of mast cells to express collagenase-1. The results of this study demonstrated firstly, the abundance of mast cells in diseased tissue

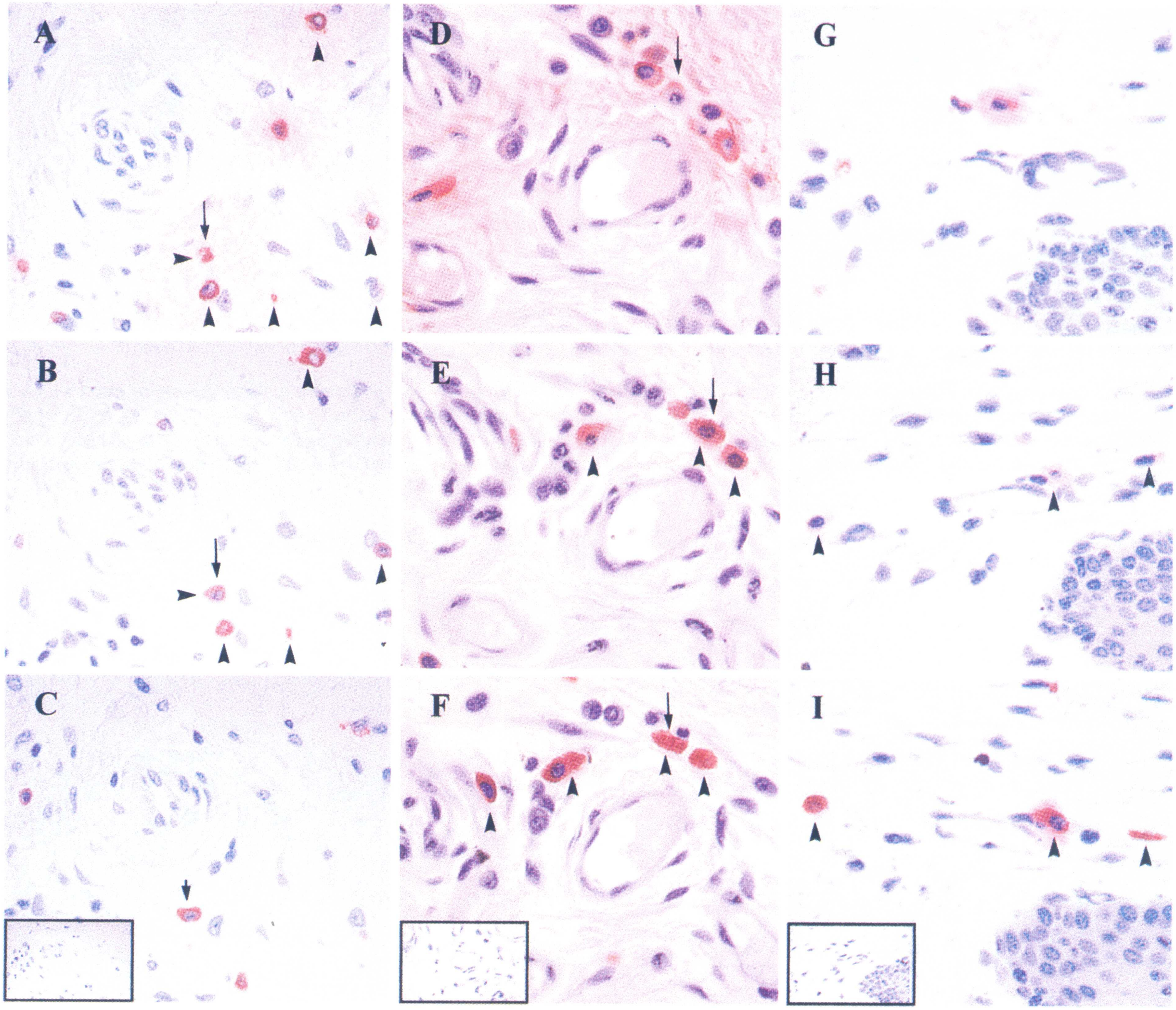

FIGURE 1 In vivo expression of collagenase-1 by human mast cells. Serial (4μm) sections of diseased human synovial tissue (A-C), pterygium tissue (D-F) and normal human conjunctiva (G-I) were immunohistochemically analyzed for the expression of tryptase (A, D, G), collagenase-1 (B, E, H), chymase (C, F, I). Some sections were incubated with preabsorbed anti-collagenase-1 mAb (inset C), an isotype control Ab (inset F) or in the absence of a primary Ab (inset I). Immunoreactivity is denoted by the red cytoplasmic staining, with hematoxylin establishing the background nuclear staining. Arrowheads and arrows identify the same mast cell in two or three contiguous tissue sections respectively. These results are representative of all tissue examined. Original magnification X500 for sections A-C and X640 for sections D-I (see Color Plate IX at the back of this issue)

and their typical perivascular localization (Fig 1 A-F). In contrast, fewer mast were detected in all normal tissue analyzed (Fig 1 G-I). Mast cells were identified based on the specificity of two monoclonal antibodies (mAbs) directed against tryptase and chymase, which are proteases stored in mast cells granules. Sections of synovial tissue derived from a patient with RA demonstrated intense immunoreactivity for collagenase-1 in large, round, granular cells (Fig 1B), and in irregular shaped cells resembling connective tissue fibroblasts and synovial linning macrophage-like cells (data not shown, and McCachren et al., 1990). Serial tissue sections revealed the identity of these cells as tryptase (Fig 1A) and chymase (Fig 1C) positive mast cells.

Corroborating evidence was generated using diseased ocular tissue (Fig 1 D-F), whereby collagenase-1 producing cells (Fig 1E), co-expressed chymase (Fig 1F). Similarly, tryptase (Fig 1G) and chymase (Fig 1I) positive mast cells in normal ocular tissue stained positively for collagenase-1 (Fig 1H), although the immunoreactive staining was much weaker than that observed in diseased tissue. Identical staining patterns were observed with all other diseased and normal human tissue examined. Of note was the diffuse extracellular as well as specific cell-associated staining observed with the tryptase Ab. This pattern of staining has previously been observed in our laboratory (Gotis-Graham and McNeil, 1997; Gotis-Graham et al., 1998), and is thought not to be associated with mast cell degranulation. No staining was observed when tissue sections were incubated with pre-absorpted collagenase-1 mAb (Fig 1C, inset), an isotype control Ab (Fig 1F, inset), or when the primary Ab was omitted (Fig 1I, inset).

### Collagenase-1 mRNA Is Localized To Mast Cells In Human Tissue

*In situ* hybridization using a digoxigenin-labeled riboprobe on human pterygium (Fig 2A) and other diseased and normal tissue (micrographs not shown) demonstrated specific cytoplasmic hybridization signal for collagenase-1 mRNA transcripts in toluidine blue positive mast cells (Fig 2B). Additional hybridization signal for this transcript was observed in resident connective tissue cells (data not shown). Sections hybridized with the corresponding sense probe resulted in no signal (Fig 2A, inset). Examination of all tissues for TIMP-1 mRNA by ISH resulted in the absence of this transcript from mast cells using this method (data not shown).

### Collagenase-1 Is Produced By Cultured Human Mast Cells (HMC-1)

Although HMC-1 cells are an immature and a malignant mast cell line, to our knowledge this is the only human mast cell line available which may best represent mast cells *in vivo*. As previously shown (Butterfield et al., 1988), and as demonstrated in Figure 3, these cells contain distinct granular immunoreactivity for tryptase (Fig 3A) but not chymase (A, inset). Like their *in vivo* counterparts, HMC-1 expressed cytoplasmic collagenase-1 (Fig 3B) and some cells contained gelatinase B (Fig 3C) and TIMP-1 (Fig 3D) immunoreactivity. TIMP-2 and –3 could not be detected by this method (micrographs not shown).

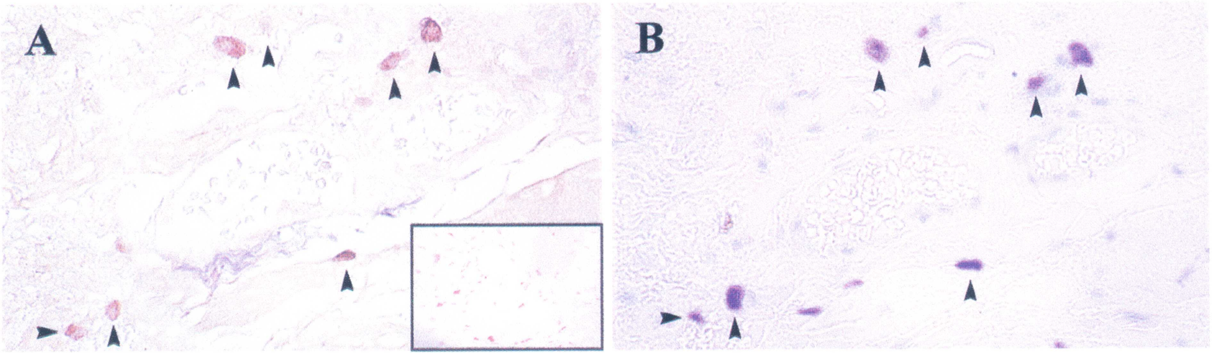

FIGURE 2 Human mast cells express collagenase-1 mRNA. Sections of pterygium tissue were hybridized with a digoxigenin-labeled collagenase-1 antisense (A) or sense (inset A) riboprobe. An adjacent section was stained with toluidine blue (B) to detect all mast cells. Hybridization signal is denoted by the blue/purple cytoplasmic staining and neutral red distinguishes the cell nuclei. Arrowheads identify the same mast cell in two sequential tissue sections. Similar results were obtained with all other diseased and normal tissue examined. Original magnification X313 (see Color Plate X at the back of this issue)

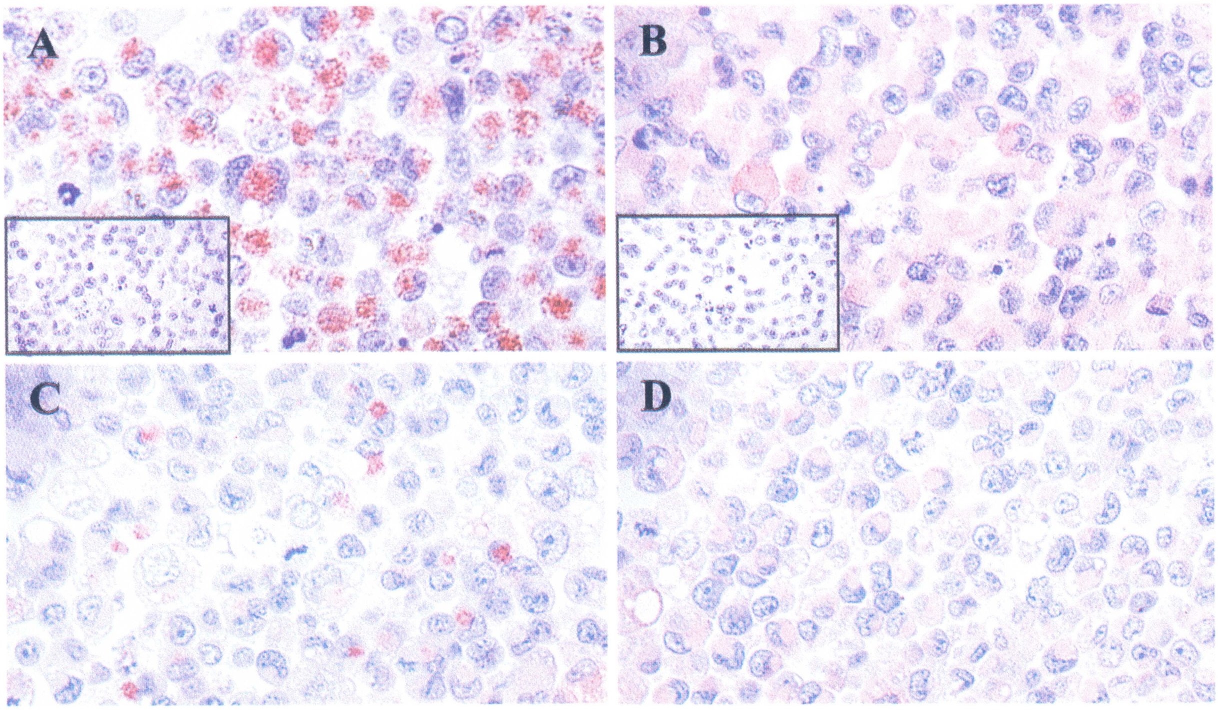

FIGURE 3 HMC-1 cells express collagenase-1, gelatinase B, and TIMP-1. HMC-1 cells were cultured, pelleted, fixed, and sectioned for histochemical analysis using an anti-tryptase mAb (A), anti-chymase Ab (inset A), anti-collagenase-1 mAb (B), anti-gelatinase B mAb (C) or an anti-TIMP-1 mAb (D). When an isotype control Ab was used, no immunoreactivity was observed (inset B). Immunoreactivity is denoted by the red cytoplasmic staining. Cells were counterstained with hematoxylin. This data is representative of four separate experiments. Original magnification X500 (see Color Plate XI at the back of this issue)

### Cell-To-Cell Contact Induces Collagenase-1 Production

To determine whether cell-to-cell contact induced collagenase-1 secretion, HMC-1 cells were co-cultured with human scleral fibroblasts (HSF) (cells that express little collagenase-1 even when stimulated with pro-inflammatory cytokines) (Di Girolamo et al., 1995). In this set of experiments, HSF were allowed to grow to semiconfluence, after which HMC-1 cells were added. Conditioned media (CM) derived from co-culture experiments displayed increased collagenase-1 immunoreactivity (Fig 4, lane 3). In contrast, CM derived from HSF or HMC-1 alone demonstrated little or no reactivity for this proteinase (Fig 4, lanes 2 and 4 respectively).

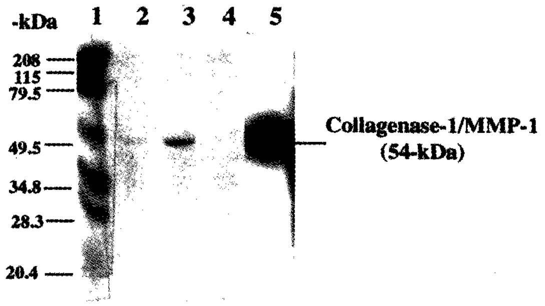

FIGURE 4 Induction of collagenase-1 in co-cultures. Human scleral fibroblasts were cultured to semi-confluence and co-cultured with HMC-1 cells. Supernatants from the scleral fibroblasts cultured alone (lane 2), HMC-1 cultured alone (lane 4), co-cultures (lane 3) and CM from PMA stimulated human synovial fibroblasts (lane 5) were analysed by immunoblot using a collagenase-1 mAb. This blot is representative of three separate experiments

### HMC-1 Cells Secrete Collagenase-1

HMC-1 were cultured under serum-free conditions over 48 hrs., afterwhich the CM was prepared for immunoblotting. HMC-1 cells (Fig 5, lane 2) constitutively produced collagenase-1. Increased immunoreactivity for this enzyme was observed in the CM of PMA stimulated mast cells (Fig 5, lane 1). Interestingly, the addition of A23187 (degranulating agent) resulted in no increase in the intensity of the 54-kDa immunoreactive band compared to control levels (Fig 5, lane 3). These results suggest that collagenase-1 is not stored in mast cell granules but secreted upon synthesis. The 54-kDa band detected in HMC-1 CM co-migrated precisely with the previously characterized collagenase-1 derived from human synovial fibroblasts (Fig 5, lane 4). These data also confirm the specificity of the collagenase-1 mAb used in Figures 1, 3, and 4.

### PMA Induces Gelatinolytic Activity in Mast Cell Lines

Gelatin-substrate zymography is a powerful technique, which allows for the detection of gelatinases from different mammalian species in CM samples. This arm of the study was initiated to determine the gelatinolytic profile of HMC-1 cells. No gelatinolytic bands were found in the supernatants from unstimulated cells (Fig 6, lane 2). However, exposure to PMA (Fig 6, lane 3) resulted in the induction of a prominent gelatinolytic band which migrated to 92-kDa, degraded the gelatin substrate and co-migrated with gelatinase B produced by the human fibrosarcoma cell line (HT1080) (Fig 6, lane 6). Gelatinase A was not detected in HMC-1 supernatants. In parallel, CM from the dog mast cells (BR), previously shown to contain gelatinolytic activity (Fang et al., 1996), displayed an increased area of clearance in the zymogram (Fig 6, lanes 4 & 5). CM from unstimulated BR cells contained gelatinase A & B-like bands which co-migrated with gelatinase A (72-kDa) and gelatinase B (92-kDa) produced by HT1080 cells. Cells treated with PMA secreted higher levels of both pro- and active gelatinase A and B-like activities, (as shown by a decrease in MW of approximately 10-kDa) (Fig 6, lane 5). Gels loaded with the identical samples but incubated in substrate buffer containing EDTA or 1, 10-phenanthroline (potent inhibitors of MMPs), resulted in no lytic activity (data not shown), suggesting that the bands displayed on the zymogram were derived from MMPs and not from serine proteinases. In addition, their migratory pattern and their potent activity against gelatin were other criteria used to identify and classify these enzymes as MMPs.

### TIMP-1 And –2 But Not TIMP-3 mRNAs Are Expressed By HMC-1

Although no immunoreactivity for the TIMPs was observed in mast cells in any human tissue examined, some immunoreactivity for TIMP-1 was observed in HMC-1 cells (Fig 3D). The sensitive technique of RT-PCR was employed to determine the expression of TIMPs in HMC-1. Of the three TIMPs analyzed, HMC-1 cells expressed TIMP-1 and –2 mRNAs (Fig 7A & B respectively). Interestingly, these two transcripts were differentially regulated, as TIMP-1 mRNA was apparently down-regulated by PMA (Fig 7A, lane 2), whereas TIMP-2 was induced by PMA (Fig 7B, lane 2). TIMP-3 mRNA was not detected by HMC-1 cells (Fig 7C), but was in human scleral fibroblasts (HSF) (Fig 7C, lane 3). Amplification of GAPDH mRNA (Fig 7D) was identical between control and PMA stimulated HMC-1 cells, providing an appropriate control.

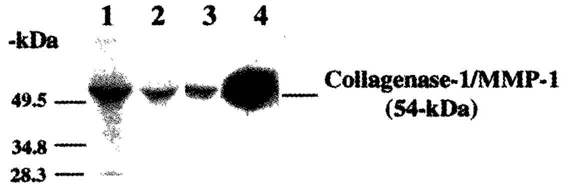

FIGURE 5 HMC-1 cells secrete collagenase-1. CM from PMA stimulated (lane 1), control (lane 2), and A23187 exposed (lane 3) HMC-1 cells and supernatants from PMA treated human synovial fibroblasts (lane 4) were analysed by Western immunoblotting using a collagenase-1 mAb. A single immunoreactive band migrating to approximately 54-kDa was observed. This blot is representative of three individual experiments

### DISCUSSION

Mast cells have been shown to participate in a range of inflammatory and immunological events such as acute immediate hypersensitivity reactions, host defence against parasites, angiogenesis, wound repair and fibrosis, and connective tissue turnover (McNeil 1996). Such processes are partially mediated by the large range of bioactive molecules released by these cells upon activation and degranulation. Along with the growing list of cytokines, chemokines (Tedla et al., 1998; Wang et al., 1998; Moller et al., 1993) and an arsenal of serine proteases stored by mast cells, the current study has identified a class of enzymes active against specific matrix components. Evidence of MMP production by human mast cells was confirmed in both diseased and normal tissue and in a human mast cell line. To our knowledge this is the first demonstration of the production of MMPs by human mast cells *in vivo*.

The localization of interstitial collagenase to mast cells has particular significance, as this enzyme specifically denatures interstitial collagen type-I, II, and III, matrix proteins frequently encountered by mast cells as they migrate through the connective tissue. Recently, it has been shown that other granulocytes such as eosinophils use MMPs to migrate through basement membranes (Okada et al., 1997) and gelatinase B has been shown to play a role in neutrophil migration (Declaux et al., 1996). Alternatively, the production of MMPs by mast cells could contribute to the extensive tissue degradation in diseases such as RA (Tetlow and Woolley, 1995; Gotis-Graham and McNeil, 1997), cancer (Dabbous et al., 1986), asthma (Galli, 1993), inflammatory ocular diseases (Di Girolamo et al., 1998a; Di Girolamo et al., 1998b), and in the normal remodelling which occurs in the human endometrium (Salamonsen and Woolley, 1996). It is interesting to note that these previous studies failed to identify MMPs in human mast cells. This discrepancy could have been due to the source of primary Ab, as the tissue fixative was generally similar to that used in present study. The specificity of the collagenase-1 mAb used in the current study was verified by antibody/antigen preabsorption (Fig 1C, inset), and Western blotting (Figs 4 & 5), where a single immunoreactive species which migrated to approximately 54-kDa was observed. Corroborating evidence has recently been presented by several investigators who have localized stromelysin-1 (MMP-3) to murine mast cells (Brownell et al., 1995) and gelatinase B to dog mast cells (Fang et al., 1996). There is however one study which disputes the results of the present study, and suggests that interstitial collagenase actually binds mast cell granules (Krejci et al., 1992). The authors of that particular study added exogenous pro- or active collagenase-1 to frozen or paraffin-embedded sections of normal, malignant and other pathological human tissue and found that collagenase-1 bound (via heparin) exclusively to mast cells and mast cell granules upon degranulation, as opposed to mast cells themselves producing this enzyme. With respect to the present study, it is unlikely that mast cells have bound collagenase-1 from the microenvironment, as firstly the immunohistochemical data demonstrated that the immunoreactive staining was cytoplasmic and not membrane associated (Fig 1B, E, H). In addition, there was no evidence of degranulation in the tissue sections analyzed. Secondly, mast cells expressed collagenase-1 mRNA transcripts (Fig 2A), and thirdly, HMC-1 cells secreted detectable levels of collagenase-1.

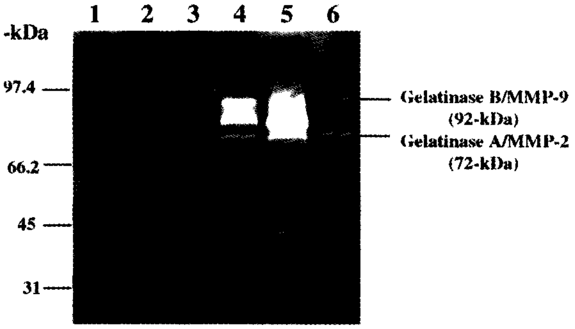

FIGURE 6 HMC-1 cells secrete gelatinase B. HMC-1 cells (lanes 2 & 3), canine BR mast cells (lanes 4 & 5) and HT1080 (lane 6) were cultured in serum-free media under control conditions (lanes 2, 4, 6) or stimulated with PMA (lanes 3 & 5) and the CM analyzed by gelatin zymography. A low MW standard was run in parallel (lane 1). This data is representative of four experiments

TABLE I Primer pairs used for PCR analysis

| | | |
|---|---|---|
| (a) TIMP-1+ | F | 5¹-TGC ACC TGT GTC CCA CCC CAC CCA CAG ACG-3¹ |
| | R | 5¹-GGC TAT CTG GGA CCG CAG GGA CTG CCA GGT-3¹ |
| (b) TIMP-2Δ | F | 5¹-GCA GAT GTA GTG ATC AGG GC-3¹ |
| | R | 5¹-TAT GGG TCC TCG ATG ACG AG-3¹ |
| (c) TIMP-3⊥ | F | 5¹-CCA TCA AGC AGA TGA AGA TGT ACC-3¹ |
| | R | 5¹-GGT AGT AGC AGG ACT TGA TCT TGC-3¹ |
| (d) GAPDH* | F | 5¹-TGA TGA CAT CAA GAA GGT GGT GAA G-3¹ |
| | R | 5¹-TCC TTG GAG GCC ATG TGG GCC AT-3¹ |

**Abbreviations**: GAPDH; Glyceraldehyde 3 phosphate dehydrogenase, F; Forward primer, R; Reverse primer. Previous studies (+ Di Girolamo et al., 1998b; Δ Stetler- Stevenson et al., 1990; ⊥ Kenney et al., 1998; *Schimonovitz et al., 1994) have successfully used these primer sets to amplify the respective gene targets.

The function of mast cell serine proteases chymase and tryptase are yet to be fully elucidated, although they have been shown to have differing substrate specificity. Saarinen et al (1994) have recently demonstrated the specific cleavage of procollagenase-1 by human mast cells chymase, and similar data was presented by Suzuki et al (1995) who showed that rat mast cell proteinase II (an enzyme equivalent to human chymase) activated procollagenase-1. The Western blotting data presented in the current study showed that the collagenase-1 secreted by HMC-1 cells was present in the latent or zymogen form. This is not surprising since HMC-1 cells do not express chymase. It is however tempting to speculate that the collagenase-1 secreted by human chymase positive mast cells *in vivo*, may be promptly activated upon degranulation. This was recently demonstrated by Fang et al (1996) who showed that not only were dog mast cells capable of producing gelatinase B, but this enzyme could be activated by chymase derived from the same cells after degranulation. Similarly, we have previously demonstrated that HMC-1 cells are capable of producing stromelysin-1, and that supernatants derived from these cells contained active enzyme (Di Girolamo et al., 1998a). It was speculated that mast cell derived products (possibly tryptase) were contributing to this activation.

Whereas previous reports have indicated that mast cell derived serine proteases are capable of activating MMPs produced by other cells, the data presented in the current study suggest that human mast cells are capable of producing both families of enzymes. Therefore, it is tempting to speculate that the serine proteases released by mast cells upon degranulation may function to activate collagenase-1 produced by the same cell. The nett effect may be localized tissue damage in diseased states. Alternatively, under normal physiological conditions, MMPs may be required for the passive migration of mast cells through connective tissues. Data supporting this hypothesis was demonstrated in Figure 1H, where it was apparent that the staining intensity for collagenase-1 in mast cells was diminished in normal compared to diseased human tissue. Similarly, collagenase-1 production was shown to be induced in HMC-1 cells. Future studies will be aimed at determining the relative contribution of MMPs by mast cells versus other inflammatory and resident connective tissue cells. In summary, our results imply that mast cells play a critical role in tissue destruction and matrix turnover in both pathological and physiological conditions.

### MATERIALS AND METHODS

### Diseased And Normal Human Tissue

Synovial tissue specimens from patients with rheumatoid arthritis (n=6), human pterygium specimens (n=6), inflamed tonsilar tissue (n=4), normal synovium (n=3), normal conjunctiva (n=8), and normal small bowel (n=3) were obtained from archival or

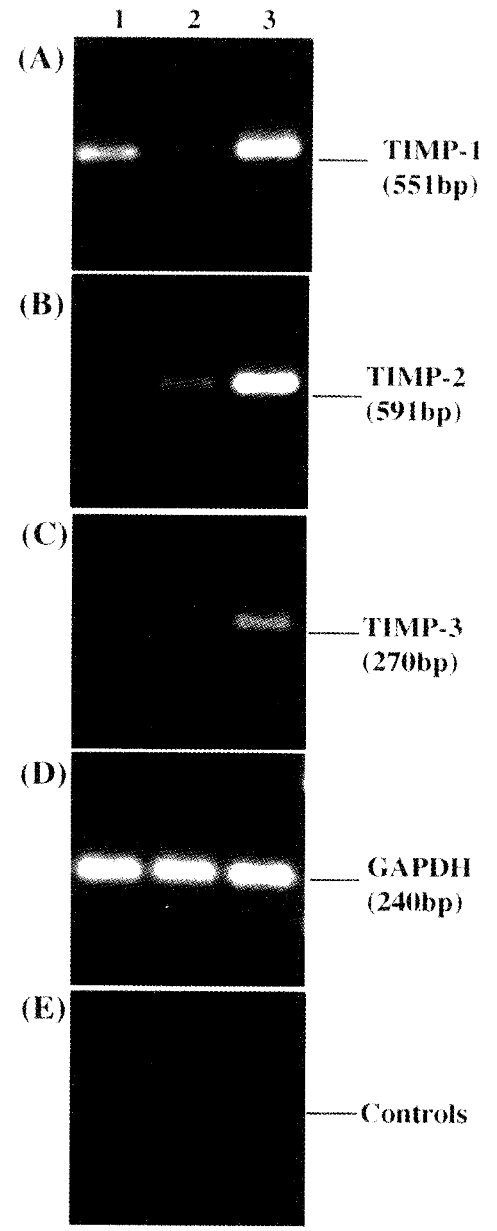

FIGURE 7 RT-PCR analysis for TIMP mRNA expression in HMC-1 cells. Total RNA was extracted from HMC-1 cells (lane 1 & 2) and from HSF (lane 3) under control conditions (lanes 1 & 3) or after exposure to PMA (lane 2). In the presence of reverse transcriptase and gene specific primers for TIMP-1 (A), TIMP-2 (B), TIMP-3 (C) and GAPDH (D), products of predicted size were formed. When no TIMP-1 primers were included (E lane 1), no reverse transcriptase was used (E lane 2) and no template was added (E lane 3), no PCR product formed. Product size was estimated with a 100bp ladder (Gibco BRL). These results were obtained in two other experiments postmortem material held in the Department of Anatomical Pathology, The Prince of Wales Hospital, and the Department of Eye Pathology, Sydney Eye Hospital, Sydney, Australia.

### Preparation Of RNA Probes And In Situ Hybridization

Plasmid cDNAs for human interstitial collagenase-1 and TIMP-1 were manipulated to generate digoxigenin-labeled sense and antisense collagenase-1 (530bp) and TIMP-1 (551bp) riboprobes as previously described (Di Girolamo et al., 1995; Di Girolamo et al., 1997; Di Girolamo et al., 1998b). Non-isotopic *in situ* hybridization (ISH) was performed as previously described using these probes on 4μm sequential tissue sections (Di Girolamo et al., 1995; Di Girolamo et al., 1997; Di Girolamo et al., 1998b).

### Immunohistochemical Analysis

Diseased and control tissue was cut (2–4μm), mounted, dried, and processed for immunohistochemistry (IHC). Sections were de-paraffinized, quenched for endogenous peroxidase as previously described (Di Girolamo et al., 1998b), and incubated with a 1:5 dilution of pre-immune goat serum for 30 min. Mouse primary mAbs for human tryptase, isotype control $IgG_1$ (Dako, Carpinteria, CA), a biotinylated human chymase (Chemicon, Temecula, CA), human collagenase-1, gelatinase B, and TIMP-1 (ICN Biochemicals, Sydney, Australia) were incubated with tissue sections for 30 min., after which a 1:200 dilution of a biotinylated secondary goat anti-mouse Ab was applied for 30 min. Sections were extensively washed in TBS and a 1:100 dilution of HRP-conjugated streptavidin (Dako Corp, Carpinteria, CA) was added for 60 min., followed by the addition of 3-amino-9-ethylcarbazole (AEC; Sigma, Sydney, Australia). Sections were counterstained with hematoxylin, viewed under light microscope and photographed.

### Metachromatic Staining

Tissue sections were prepared as per IHC, except that after dewaxing each section was treated with 0.5N HCl for 10 min. then stained for 90 min. with a solution consisting of 1% toluidine blue (BDH Chemicals, Sydney, Australia) in 0.5N HCl. Sections were not counter-stained but washed in water and briefly de-hydrated through increasing grades of alcohol. This staining procedure was used as an alternative method for detecting mast cells in human tissue.

### Cell Culture

The human mast cell line HMC-1 was a generous gift from Dr J.H. Butterfield (Mayo Clinic, Rochester, MN, USA) and the canine mast cell line BR was a kind gift from Dr K.C. Fang (CVRI, UCSF, CA, USA). These cells were cultured in 75cm$^2$ tissue flasks (Nunc, Roskilde, Denmark) in RPMI (Trace Biosciences, Sydney, Australia) supplemented with 10% FBS (Trace Biosciences) and 100 Units/mL penicillin and 100 µg/mL streptomycin (Trace Biosciences). All cell culture media and solutions were filtered to minimize endotoxin as previously described (Di Girolamo et al., 1997; Di Girolamo et al., 1998b). Cells were extensively washed in PBS, counted and seeded at $2.5 \times 10^6$cells/mL in 75cm$^2$ flasks in serum-free media (SFM; 0.2% BSA/EMEM) with or without 10 ng/mL PMA (Sigma, Sydney, Australia) or treated with A23187 (Sigma). For co-culture experiments, human scleral fibroblasts (HSF) were grown to semi-confluence (each flask containing approximately $2 \times 10^6$ cells), extensively washed with PBS and $2 \times 10^6$ HMC-1 cells added. For some experiments, CM and RNA was harvested after 48 hrs. and stored in aliquots at –70°C until used in further analyses. Other cells used in the present study included: human synovial fibroblasts from a patient with RA and the human fibrosarcoma cell line HT1080 (American Tissue Culture Collection, Rockville, MD), cells known to secrete several species of MMPs and TIMPs.

### Extraction Of RNA And RT-PCR Analysis

Total RNA was extracted as previously described (Di Girolamo et al., 1997; Di Girolamo et al., 1998b). Reverse transcription was performed according to the manufacturer's instructions, using the "Preamplification System for First Strand cDNA Synthesis Kit" (Gibco BRL, Gaithersburg, MD). Aliquots (1µL) of cDNA were amplified by PCR using 100nM each of the forward and reverse gene specific primer (GSP) (see Table I), using similar conditions to those previously described (Di Girolamo et al., 1998b). Semi-quantitative PCR was established by terminating reactions at regular intervals of 10, 15, 20, 25, 30, and 35 cycles for each primer pair to ensure that the products formed were within the linear portion of the amplification curve. PCR conditions of temperature, cycle number and length, and restriction enzyme digestions were performed precisely as described previously (Di Girolamo et al., 1998b). Products were visualized on 1.2% agarose gels stained with ethidium bromide.

### SDS-PAGE Gelatin-Substrate Zymography

Gelatin-substrate zymography was performed as previously described (Di Girolamo et al., 1997; Di Girolamo et al., 1998b). Some gels were incubated with 1, 10-phenanthroline (1mmol/L) or EDTA (10mmol/L) (Sigma), both potent MMP inhibitors.

### Western Blot Analysis

Western blotting was performed as previously described (Di Girolamo et al., 1997; Di Girolamo et al., 1998a; Di Girolamo et al., 1998b) using a mouse anti-human collagenase-1 mAb (ICN Biochemicals, Sydney, Australia). Membranes were placed in a chemiluminescent reagent for non-radioactive detection of proteins (Dupont, Sydney, Australia) to amplify the immunoreactive signal, then exposed to X-ray film.

### *References*

Brownell E., Fiorentino L., Jolly G., Wolfe K., Kincaid S., Seperack P., and Visco D. (1995) Immunolocalization of stromelysin-related protein in murine mast cell granules. Int Arch Allergy Immunol 107:333–335.

Butterfield J.H., Weiler D., Dewald G., and Gleich G.J. (1988) Establishment of an immature mast cell line from a patient with mast cell leukemia. Leukemia Res 12:345–355.

Dabbous M. Kh., Walker R., Haney L., Carter L.M., Nicholson G.L., and Woolley D.E. (1986) Mast cells and matrix degradation at sites of tumour invasion in rat mammary adencarcinomas. Br J Cancer 54:459–465.

Declaux C., Delacourt C., d'Ortho M.-P., Boyer V., Lafuma C., and Harf A. (1996) Role of gelatinase B and elastase in human polymorphonuclear neutrophil migration across basement membrane. Am J Respir Cell Mol Biol 14:288–295.

Di Girolamo N., McCluskey P.J., Lloyd A., and Wakefield D. (1995) Stromelysin (matrix metalloproteinase-3) and tissue inhibitor of metalloproteinase (TIMP-1) mRNA expression in scleritis. Ocular Immunol Inflam 3:181–194.

Di Girolamo N., Lloyd A., McCluskey P., Filipic M., and Wakefield D. (1997) Increased expression of matrix metalloproteinases in vivo in scleritis tissue and in vitro in cultured human scleral fibroblasts. Am J Pathol 105:653–666.

Di Girolamo N., McCluskey P., Lloyd A., and Wakefield D. (1998a) Matrix metalloproteinases and mast cell tryptase in scleritis. In Uveitis Today Ohno S., Aoki M., Usui M., Uchio E. eds (Tokyo: Elsevier Science), pp. 103–105.

Di Girolamo N., Tedla N., Lloyd A., and Wakefield D. (1998b) Expression of matrix metalloproteinases by human plasma cells and B lymphocytes. Eur J Immunol 28:1773–1784.

Fang K.C., Raymond W.W., Lazarus S.C., and Caughey G.H. (1996) Dog mastocytoma cells secrete a 92-kD gelatinase activated extracellularly by mast cell chymase. J Clin Invest 97:1589–1596.

Galli S.J. (1993) New concepts about the mast cell. N Engl J Med 328:257–265.

Godfrey H.P., Ilardi C., Engber W., and Graziano F.M. (1984) Quantitation of human synovial mast cells in rheumatoid arthritis and other rheumatic diseases. Arthritis Rheum 27:852–856.

Gordon J.R., and Galli S.J. (1990) Mast cells as a source of both preformed and immunologically inducible TNF-a/cachetin. Nature 346:274–276.

Gotis-Graham I. and McNeil H.P. (1997) Mast cell responses in rheumatoid synovium. Association of the $MC_{TC}$ subset with matrix turnover and clinical progression. Arthritis Rheum 40:479–489.

Gotis-Graham I., Smith M.D., Parker A., and McNeil H.P. (1998) Synovial mast cell responses during clinical improvement in early rheumatoid arthritis. Ann Rheum Dis 57:664–671.

Gruber S.L., Marchese M.J., Suzuki K., Schwartz L.B., Okada Y., Nagase H., and Ramamurthy N.S. (1989) Synovial procollagenase activation by human mast cell tryptase: dependence upon matrix metalloproteinase 3 activation. J Clin Invest 84:1657–1662.

Guedez L., Stetler-Stevenson W.G., Wolff L., Fukushima P., Mansoor A., and Stetler-Stevenson M. (1998) In vitro suppression of programmed cell death of B cells by tissue inhibitor of metalloproteinase-1. J Clin Invest 102:2002–2010.

Johnson J.L., Jackson C.L., Angelini G.D., and George S.J. (1998) Activation of matrix-degrading metalloproteinases by mast cell proteases in atherosclerotic plaques. Arterioscler Thromb Vasc Res 18:1707–1715.

Kaartinen M., Van de wal A.C., Van der loos C.M., Piek J.J., Koch K.T., Becker A.E., and Kovanen P.T. (1998) Mast cell infiltration in acute coronary syndromes: implications for plaque rupture. J Am Coll Cardiol 32:606–612.

Kenney M.C., Chwa M., Alba A., Saghizadeh M., Huang Z.-S., and Brown D.J. (1998) Localization of TIMP-1, TIMP-2, TIMP-3, gelatinase A and gelatinase B in pathological human corneas. Curr Eye Res 17:238–246.

Krejci N.C., Maldonado-Knapp D., Rudd R.J., Bauer E.A., and McGuire J. (1992) Dermal mast cell granules bind interstitial procollagenase and collagenase. J Invest Dermatol 98:748–752.

Lohi J., Harvima I., and Keski-Oja J. (1992) Pericellular substrates of human mast cell tryptase: 72,000 dalton gelatinase and fibronectin. J Cell Biochem 50:337–349.

Mauviel A. (1993) Cytokine regulation of metalloproteinase gene expression. J Cell Biochem 53:288–295.

McCachren S.S., Haynes B.F., and Niedel J.E. (1990) Localization of collagenase mRNA in rheumatoid arthritis synovium by in situ hybridization histochemistry. J Clin Immunol 10:19–27.

McNeil H.P. (1996) The mast cell and inflammation. Aust NZ J Med 26:216–225.

Moller A., Lippert U., Lessmann D., Kolde G., Hamann K., Welker P., Schadenforf D., Rosenbach T., Luger T., and Czarnetzki B.M. (1993) Human mast cells produce IL-8. J Immunol 151:3261–3266.

Okada S., Kita H., George T.J., Gleich G.J., and Leiferman K.M. (1997) Migration of eosinophils through basement membrane components *in vitro*: role of matrix metalloproteinase-9. Am J Respir Cell Mol Biol 17:519–528.

Powers M.R., Qu Z., Obrien B., Wilson D.J., Thompson J.E., and Rosenbaum J.T. (1997) Immunolocalisation of bFGF in pterygia: association with mast cells. Cornea 16:545–549.

Ries C., and Petrides P.E. (1995) Cytokine regulation of matrix metalloproteinase activity and its regulatory dysfunction in disease. Biol Chem 376:345–355.

Saarinen J., Kalkkinen N., Welgus H.G., and Kovaven P.T. (1994) Activation of human interstitial procollagenase through direct cleavage of the $Leu^{83}$-$Thr^{84}$ bond by mast cell chymase. J Biol Chem 269:18134–18140.

Salamonsen L.A., and Woolley D.E. (1996) Matrix metalloproteinases in normal menstruation. Human Reprod 11:(Suppl 2)124–133.

Schwartz L.B. (1994) Tryptase: a mast cell serine protease. Methods Enzymol 244:88–100.

Shimonovitz S., Hurwitz A., Dushnik M., Anteby E., Geva-Eldar T., and Yagel S. (1994) Developmental regulation of the expression of 72 and 92 kd type IV collagenases in human trophoblasts: a possible mechansim for control of trophoblast invasion. Am J Obstet Gynecol 171:832–838.

Stetler-Stevenson W.G. (1996) Dynamics of matrix turnover during pathologic remodeling of the extracellular matrix. Am J Pathol 148:1345–1350.

Stetler-Stevenson W.G., Bersch N., and Golde D.W. (1992) Tissue inhibitor of metalloproteinase-2 (TIMP-2) has erythroid-potentiating activity. FEBS 296:231–234.

Stetler-Stevenson W.G., Brown P.D., Onisto M., Levy A.T., and Liotta L.A. (1990) Tissue inhibitor of metalloproteinase-2 (TIMP-2) mRNA expression in tumor cell lines and human tumor tissues. J Biol Chem 265:13933–13938.

Suzuki K., Lees M., Newlands G.F., Nagase H., and Woolley D.E. (1995) Activation of precursors for matrix metalloproteinases 1 (interstitial collagenase) and 3 (stromelysin) by rat mast-cell proteinases I and II. Biochem J 305:301–306.

Tedla N., Wang H.-W., McNeil H.P., Di Girolamo N., Hampartzoumian T., Wakefield D., and Lloyd A. (1998) Regulation of T lymphocyte trafficking into lymph nodes during an immune response by the chemokines, MIP-1α and MIP-1β. J Immunol 161:5663–5672.

Tetlow L.C., and Woolley D.E. (1995) Mast cells, cytokines, and metalloproteinases at the rheumatoid lesion: dual immunolocalisation studies. Ann Rheum Dis 54:896–903.

Vartio T., Seppa H., and Vaheri A. (1981) Susceptibility of soluble and matrix fibronectins to degradation by tissue proteases, mast cell chymase and cathepsin G. J Biol Chem 256:471–477.

Wang H.-W., Tedla N., Lloyd A.R., Wakefield D., and McNeil H.P. (1998) Mast cell activation and migration to lymph nodes during induction of an immune response in mice. J Clin Invest 102:1617–1626.

Woolley D.E., Bartholomew J.S., Taylor D.J., and Evanson J.M. (1989) Mast cells and rheumatoid arthritis. In: Mast cell and basophil differentiation and function in health and disease, Galli S.J., and Austen F.K., Eds (New York: Raven Press), pp. 183–193.

